# Demographics and Use of an Addiction Helpline for Concerned Significant Others: Observational Study

**DOI:** 10.2196/55621

**Published:** 2025-04-14

**Authors:** Rachel Chernick, Amanda Sy, Sarah Dauber, Lindsey Vuolo, Bennett Allen, Fred Muench

**Affiliations:** 1 Partnership to End Addiction New York, NY United States; 2 Department of Population Health NYU Grossman School of Medicine New York, NY United States

**Keywords:** family, hotline, helpline, warmline, crisis continuum, substance abuse, addiction, youth, concerned significant other, digital behavioral health, parents, substance use disorders, well-being, public health intervention, cannabis, treatment, opioids, men, women, assessments

## Abstract

**Background:**

Concerned significant others (CSOs) play a significant role in supporting individuals with substance use disorders. There is a lack of tailored support services for these CSOs, despite their substantial contributions to the well-being of their loved ones (LOs). The emergence of helplines as a potential avenue for CSO support is outlined, culminating in the focus on the Partnership to End Addiction’s helpline service, an innovative public health intervention aimed at aiding CSOs concerned about an LO’s substance use.

**Objective:**

The article analyzes the demographics and use patterns of the Partnership to End Addiction’s helpline service, highlighting the critical role of such services, and advocating for expanded, tailored support models.

**Methods:**

This observational study draws data from 8 data platforms spanning April 2011 to December 2021, encompassing 24,096 client records. Surveys were completed by helpline specialists during synchronous telephone calls or self-reported by CSOs before helpline engagement. Collected information encompasses demographics, interaction language, substance of concern, CSO-LO relationship, and the LO’s “use state,” that is, their location on the continuum of substance use.

**Results:**

CSOs primarily comprised women (13,980/18,373, 76.1%) seeking support for their children (1062/1542, 68.9%). LOs were mostly male (1090/1738, 62.7%), aged 18-25 years (2380/7208, 33%), with primary substance concerns being cannabis (5266/12,817, 40.9%), opioids (2445/12,817, 19%), and stimulants (1563/12,817, 12.1%). CSOs primarily sought aid for LOs struggling with substances who were not in treatment (1102/1753, 62.9%). The majority of CSOs were looking for support in English (14,738/17,920, 82.2%), while the rest (3182/17,920, 17.8%) preferred to communicate in Spanish. Spanish-speaking CSOs were significantly more likely to call about cannabis (n=963, 53.7% vs n=4026, 38.6%) and stimulants (n=304, 16.9% vs n=1185, 11.3%) than English-speaking CSOs (*P*<.001). On the other hand, English-speaking CSOs were more likely to be concerned about opioids than Spanish-speaking CSOs (n=2215, 21.3% vs n=94, 5.2%; *P*<.001).

**Conclusions:**

The study illuminates the helpline’s pioneering role in aiding CSOs grappling with an LO’s substance use. It highlights helplines as crucial resources for CSOs, revealing key demographic, substance-related, and use-state trends. The dominant presence of women among users aligns with other helpline patterns and reflects traditional caregiving roles. While parents form a significant percentage of those reaching out, support is also sought by siblings, friends, and other family members, emphasizing the need for assistance for other members of an LO’s social network. Spanish-speaking individuals’ significant outreach underscores the necessity for bilingual support services. Substance concerns revolve around cannabis, opioids, and stimulants, influenced by age and language preferences. The helpline serves as an essential intermediary for CSOs, filling a gap between acute crisis intervention services and formalized health care and treatment services. Overall, the study highlights this helpline’s crucial role in aiding CSOs with tailored, accessible support services.

## Introduction

### Overview

Substance misuse and its consequences remain a profound public health crisis in the United States. Over 1 million individuals (about the population of Delaware) died from overdose between 1999 and 2021 [1]. Additionally, 46.3 million people (about twice the population of New York State) aged 12 years or older had a past-year substance use disorder (SUD) in 2020 [2].

The burdens associated with SUD and overdose fall both on individuals with SUDs and their families and concerned significant others (CSOs). At least 25% of the US population has a first-degree family member with an SUD, and up to 90% of people with SUDs live at home with family or other CSOs [3,4]. Numerous studies have reported the broad impacts of SUDs on CSOs, including on marital well-being, parental competence, family functioning, financial health, and the physical and emotional well-being of CSOs [5-9].

To our knowledge, this is the first study to present the use of a helpline service dedicated to supporting CSOs who are concerned about a loved one’s (LO’s) substance use. As a novel model of service for CSOs—a group for which few SU-specific services exist—this kind of descriptive information is key to seeing how helpline services are being used and to inform future implementation.

### Significance of Families

Historically, the addiction field has considered the family to be the cause of or an “enabler” of their LO’s addiction [10,11]. A growing body of evidence shows, however, that when families are provided with support and information, they are, in turn, able to effectively support their LO, and this is associated with positive outcomes for both the CSO and the LO. Instead of seeing the family member as the cause or contributor to the LO’s addiction, growing evidence suggests that this is “an ordinary person facing a very challenging problem” [12].

At the prevention level, parent or family interventions are associated with reduced risk of adolescent substance use initiation, slowed trajectories of adolescent substance misuse, increased adult socioeconomic success, and reduced risk of mental health disorders and sexually transmitted infections [5,13,14]. Family or CSO involvement in treatment is also associated with increased LO treatment engagement and reduced LO substance use [15-21]. Families provide recovery capital to LOs through supportive and positive relationships, material support (eg, financial, housing, and transportation), monitoring of substance use, managing medications, and intervention in the event of a relapse [5,22,23]. Interventions that address an LO’s substance use also improve the mental well-being of the impacted family member [16,18,24].

Despite growing evidence of the potential benefits of family and CSO involvement in an LO’s substance use outcomes, support for CSOs remains limited [5]. They often cope with their situations in silence, alienated by stigma and experiencing isolation, shame, and helplessness about how and where to access help [9,11,12,25]. Barriers to CSO involvement include prioritization of individual models of SUD care, lack of insurance reimbursement for CSO involvement in care, and stigma [6,9,26,27]. Given payor restrictions, new models to support CSO engagement in LO substance use prevention, care, and treatment outside the existing architecture are needed. Helplines, or noncrisis education, counseling, and referral support are a potential novel strategy to support this group [28].

### Helplines

Unlike hotlines, which generally provide 24-hour crisis support and emergency intervention services, helplines generally offer noncrisis support, information, and encouragement for individuals during fixed operating hours [29]. Helplines are available for specific concerns related to mental health issues, substance use, gambling, and domestic violence as well as those designed to support specific populations such as teens, veterans, parents with infants, LGBTQ (lesbian, gay, bisexual, transgender, and queer/questioning) individuals, and seniors [28-33]. Most helplines offer information, advice, and referrals to appropriate services and treatment providers. Some offer a more clinically guided intervention using components from motivational interviewing, cognitive behavioral therapy, or Community Reinforcement and Family Training [31,34,35].

Distinguishing helpline outcomes from hotline outcomes is difficult, as much of the research fails to distinguish between the two [36]. In general, however, helpline/hotline use is associated with decreased psychological distress, improvement in well-being, increased effectiveness at addressing problems, and high levels of service satisfaction [34-39]. While most individuals contacting hotlines are calling for help with their own situation, one study found that approximately 25% of calls to the National Suicide Prevention Lifeline were from “third-party” callers (ie, CSOs) [40]. In a study of problem gambling helplines globally, 33% of those reaching out for help were CSOs [31]. In terms of CSO-specific outcomes, a recent systematic review identified consistently positive outcomes associated with helpline/hotline use, including increased well-being, self-care planning, referrals for continued support, as well as decreases in suicidality [39].

### The Partnership Helpline

The Partnership to End Addiction’s helpline service, the focus of this study, is a nationally available free service designed to serve CSOs who are concerned about an LO’s potential use, current use, or recovery from the use of a psychoactive substance. All helpline support is tailored to the individual needs of the CSO and the LO that they are concerned about, regardless of where the LO is on the continuum of use—from prevention to recovery.

Staffed by behavioral health professionals and delivered through omnichannel support (ie, phone, text, email, or Facebook Messenger), Partnership helpline specialists use evidence-based therapeutic techniques—including motivational interviewing [41], Community Reinforcement and Family Training [42], and harm reduction approaches [43]. These interventions are intended to support CSO self-care and mental well-being, with a focus on productive communication, clear boundary-setting, and improved connectedness between the CSO and the LO to improve both CSO and LO outcomes. To achieve these goals, Helpline specialists provide information on addiction, support skills that enhance communication and reward healthy behaviors, share resources on substance use care and treatment, support self-care efforts of the CSO, offer hope, and look to enhance the CSO’s self-efficacy. CSOs select their preferred communication channels through synchronous (immediate) or asynchronous (not immediate) interactions and can use the service as many times as needed without restriction.

The Partnership helpline is advertised in print, radio, TV, and social media channels—most of which are donated media. In addition, the helpline is prominently featured on the Partnership website for individuals searching for help via an organic internet search. Finally, the Partnership offers other services to this population and CSOs are referred to the helpline from these services when appropriate. While the Partnership serves any CSO who is concerned about their LO’s substance use, advertising is primarily focused on the subgroup of CSOs that are parents or caregivers.

This study is the first to share information on the helpline service during its first 10 years in operation (April 2011-December 2021). A related study describes the Partnership’s peer-to-peer support service during a portion of the same period (2014 to 2018) [16]. This study focused on a subset of parents who contacted the Partnership helpline between 2014 and 2018 and were then referred to the Partnership’s Peer Coaching service after their helpline contact. Of the 279 parents in the Peer Coaching sample, most were mothers (88%) who were concerned about their sons (69%). LOs were mostly adolescents aged 13-17 years (43%) and young adults aged 18-24 years (40%). Primary substances of concern in the Peer Coaching study included cannabis (60%), opioids (15%), and alcohol (7%).

This study is the first to present use and engagement data for a nationally available substance use-specific helpline for CSOs. To inform future service design and delivery, this study provides descriptive information on the population who contacts the Partnership’s helpline.

## Methods

### Overview

This is an observational study that examines information about a subgroup of CSOs reaching out for help from a national helpline. Data for this study are derived from 8 helpline datasets collected between April 2011 and December 2021 which represent a total of 24,096 client records, though the sample sizes vary depending on the dataset. A detailed description of the data sources and data cleaning procedures is available in the Multimedia Appendix 1. Survey responses were completed either by helpline specialists based on information provided by the CSO during synchronous phone calls or by CSO self-report via a web-based survey prior to receiving the helpline service. The helpline assessment captures information on both the CSO and LO, including demographics (eg, race/ethnicity, gender, and geographic location), the language of the interaction, the substance of concern, the CSO’s relationship with the LO, and the LO’s use state, which reflects the extent of use, and the severity of consequences associated with this use.

We defined use state as “where the LO is located on the continuum of substance use,” from the perspective of the CSO. The use state captures both quantity/frequency of use and severity of consequences associated with use. We operationalized the use state as a categorical response with five options as follows: (1) Prevention, (2) Early Use, (3) Struggling/In Treatment, (4) Struggling/Not in Treatment, and (5) Recovery. “Prevention” denotes an LO who has not used substances, where the CSO is looking for information on preventing substance use in the future. “Early Use” denotes an LO who is using substances occasionally, for example on the weekends or a few times a week, but their use is unlikely to meet a clinical threshold for SUD as defined in the DSM V-TR [44]. “Struggling/In Treatment” and “Struggling/Not in Treatment” denote LOs who would likely meet a clinical threshold for SUD, with the difference between the 2 being whether the LO is currently engaged or not engaged in SUD treatment. “Recovery” denotes an LO who is defined by the CSO as being in recovery from an SUD.

We merged and cleaned data from the 8 platforms into an aggregated dataset using Stata (StataCorp) [45]. While similar, minor variations in question phrasing and response sets exist between platforms, and some questions were not included in earlier surveys. For questions added in later data collection, we used the platform with the most comprehensive response set to satisfy the question. For qualitative responses, the response set with more specific answers would be sorted into and recorded under the more general groups. Due to privacy concerns, people reaching out to the helpline are not required to provide demographic information or complete assessments to receive support. As a result, our sample reflects only clients for whom demographic information is available, not all clients served during the period (N=35,000). In addition, assessment completion is not required to access helpline services, and some records contain missing data. Missing data were detailed in descriptive analyses. The final aggregated dataset includes 24,096 total unique responses collected between April 2011 and December 2021. Statistical analyses were calculated using IBM SPSS statistics [46]. This study uses the STROBE (Strengthening the Reporting of Observational Studies in Epidemiology) guidelines for reporting observational studies (see checklist in Multimedia Appendix 2) [47].

### Ethical Considerations

The New York University Langone Health institutional review board was contacted by the authors, and based on the self-certification documentation submitted, has determined that no institutional review board review is needed for this study as the study does not meet human subjects’ research criteria. This determination was based on the following 3 items. We are (1) not conducting an intervention, (2) not using data that includes private, identifiable information, and (3) not collecting data with identifiable information.

All service users are informed through our terms of use that deidentified data may be used for research purposes [48]. Since the data are not derived from human subjects’ research and are deidentified to ensure that individual identities cannot be ascertained, informed consent is not applicable. No compensation was provided to service users, and no specific recruitment was undertaken for this study; details about general helpline recruitment are provided above.

## Results

Results are based on 24,096 client records from the helpline registered between April 2011 and December 2021. Table 1 provides demographic information about clients contacting the helpline. The majority of the CSOs seeking support identified as women (13,980/18,373, 76.1%), with those identifying as men accounting for most of the remainder (4391/18,373, 23.9%). Callers who reported being gender nonbinary consisted of less than 1% of the sample (n=2). The vast majority of those seeking support were parents concerned about their children, accounting for 68.9% (n=1062) of the total number of those completing this question. The remainder were looking for support for a partner (149/1542, 9.7%), sibling (118/1542, 7.7%), friend (101/1542, 6.5%), parent (73/1542, 4.7%), or grandchild (39/1542, 2.5%).

**Table 1:** Demographic characteristics of helpline concerned significant others (CSOs).

Demographics		Participants, n (%)
**CSO gender^a^ (n=18,373)**		
	Girl/woman	13,980 (76.1)
	Boy/man	4391 (23.9)
	Gender nonbinary	2 (0.0)
**CSO concerned about^b^ (n=1542)**		
	Child	1062 (68.9)
	Partner	149 (9.7)
	Sibling	118 (7.7)
	Friend	101 (6.5)
	Parent	73 (4.7)
	Grandchild	39 (2.5)
**CSO region^c^ (n=17,220)**		
	Northeast	8863 (51.4)
	Southeast	2688 (15.6)
	West	2671 (15.5)
	Midwest	1670 (9.7)
	Southwest	1319 (7.7)
	US territories	9 (0.0)
**CSO language^d^ (n=17,920)**		
	English	14,738 (82.2)
	Spanish	3182 (17.8)

^a^These data are from all surveys except for Typeform and represent 18,373 individuals who responded to this question. The Typeform survey did not include a question about CSO gender and therefore was not included. The iCarol, Survey Monkey, and Formstack surveys did not include response options for a full range of gender expressions. As a result, data on individuals who identify as a gender that is not Girl/Woman or Boy/Man are underrepresented for this variable.

^b^These data are from the Typeform survey only and represent 1542 individuals who responded to this question.

^c^These data are from all 4 surveys and represent 17,220 individuals who responded to this question.

^d^These data are from all 4 surveys and represent 17,920 individuals who responded to this question.

The helpline serves any resident of the United States and US Territories. About half of the callers from this sample were based in the Northeast (8863/17,220, 51.4%). The rest were callers from the Southeast (2688/17,220, 15.6%), West (2671/17,220, 15.5%), Midwest (1670/17,220, 9.7%) and Southwest (1319/17,220, 7.7%). Less than 1% (n=9) reported being from a US territory. In terms of language, the majority of CSOs were looking for support in English (14,738/17,920, 82.2%), while the rest (3182/17,920, 17.8%) preferred to communicate in Spanish.

Table 2 provides demographic information about the LO. CSOs were largely contacting the helpline about an LO who identifies as a boy/man (1090/1738, 62.7%), with roughly a third (615/1738, 35.4%) concerned about a girl/woman. LOs who identified as gender nonbinary, transgender, or a gender not listed made up 1.8% (n=33) combined. LOs in the 18- to 25-year age group made up the largest group (2380/7208, 33%), followed closely by the 26- to 40-year age group (2131/7208, 29.6%), and then the 13- to 17-year age group (1966/7208, 27.3%). Those aged 41 years and older (519/7208, 7.2%) and the 12 or younger group (212/7208, 2.9%) were the 2 smallest groups.

**Table 2:** Demographic characteristics of the concerned significant other’s (CSO’s) loved one (LO).

Demographics		Participants, n (%)
**LO gender^a^ (n=1738)**		
	Boy/man	1090 (62.7)
	Girl/woman	615 (35.4)
	Gender nonbinary	16 (0.9)
	Transgender	12 (0.6)
	A gender not listed above	5 (0.3)
**LO age^b^ (years; n=7208)**		
	12 or younger	212 (2.9)
	13 to 17	1966 (27.3)
	18 to 25	2380 (33.0)
	26 to 40	2131 (29.6)
	41 and older	519 (7.2)

^a^These data are from the Typeform survey only and reflect 1738 individuals who responded to the question. The LO gender question was expanded to include a full range of gender identities in this survey, so earlier forms of this question contain incomplete information.

^b^These data are from the entire dataset and reflect 7208 individuals who responded to the question throughout the entire study period.

Table 3 presents 2 variables that describe the nature of the LO’s substance use: the primary substance of concern and the LO’s use state. The primary substance of concern is defined as “the substance the CSO is most concerned about” and is not necessarily the LO’s substance of choice or the substance the LO is using most often or with the greatest severity. In some cases, the CSO is not fully aware of what or how much of a substance the LO is using. In other scenarios, the CSO might be more concerned about one substance than another. The 3 most frequently reported substances of concern were cannabis (5266/12,817, 40.9%), opioids (2445/12,817, 19%), and stimulants (1563/12,817, 12.1%). These were followed by alcohol (1286/12,817, 10%), benzodiazepines (277/12,817, 2.2%), and nicotine (161/12,817, 1.3%). A little more than 10% of the sample reported they were not sure what substance their LO was using (1603/12,817, 12.4%) or that there was no single substance that was most concerning (276/12,817, 2.1%). When looking at primary substance of concern by language, Spanish-speaking CSOs were significantly more likely to call about cannabis (n=963, 53.7% vs n=4026, 38.6%): and stimulants (n=304, 16.9% vs n=1185, 11.3%) than English-speaking CSOs (*P*<.001). On the other hand, English-speaking CSOs were more likely to be concerned about opioids than Spanish-speaking CSOs (n=2215, 21.3% vs n=94, 5.2%; *P*<.001).

**Table 3:** Loved ones’ (LOs’) substance use.

LOs’ substance use				Participants, n (%)
**Primary substance of concern (for the concerned significant other)^a^ (n=12,817)**				
	Cannabis (edibles, smoking, vaping)			5266 (40.9)
	**Opioids**			2445 (19)
		Heroin	1626 (12.6)	
		Prescription painkillers (Vicodin, Oxycontin)	819 (6.4)	
	**Stimulants**			1563 (12.1)
		Cocaine or crack	479 (3.7)	
		Methamphetamine (crystal meth)	967 (7.5)	
		Prescription stimulants (Adderall, Ritalin)	117 (0.9)	
	Alcohol			1286 (10.0)
	Benzodiazepines (Klonopin, Xanax, Valium, Ativan, etc)			277 (2.2)
	Nicotine (smoking, vaping, and chewing)			161 (1.3)
	Multiple substances (I don’t have one substance that concerns me most)			276 (2.1)
	Not sure			1603 (12.4)
**LO use state^b^ (n=1753)**				
	Prevention			88 (4.9)
	Early use			327 (18.7)
	Struggling/not in treatment			1102 (62.9)
	Struggling/in treatment			129 (7.4)
	Recovery			109 (6.2)

^a^These data are from all 4 surveys and represent 12,817 individuals who responded to this question.

^b^These data are from the Typeform survey only and represent 1753 individuals who responded to this question.

The largest group were CSOs concerned about an LO who was struggling with substances and was not in treatment (1102/1753, 62.9%), with the second largest group reporting that they were concerned about an LO who was using substances occasionally (327/1753, 18.7%). The third largest group was concerned about an LO struggling with substances that were in treatment (129/1753, 7.4%), followed by the recovery (109/1753, 6.2%) and prevention groups (88/1753, 4.9%).

## Discussion

### Principal Findings

To our knowledge, this is the first study illustrating helpline use specifically designed to support a CSO reaching out about the substance use of an LO. Results show that families are interested in helpline support and that when they are aware of this support, they will use it. The dominant presence of women among these helpline users aligns with other helpline services, reflecting traditional caregiving roles. While parents form a significant percentage of those reaching out, support is also sought by siblings, friends, and other family members, emphasizing the need for assistance for other members of an LO’s social network. Spanish-speaking individuals’ significant outreach underscores the necessity for bilingual support services due to language and cultural barriers among these CSOs. Substance concerns revolve around cannabis, opioids, and stimulants, influenced by age and language preferences. Findings from this study underscore the critical role of helplines in providing support for CSOs grappling with the substance use of their LOs.

### CSO Demographics

Women were the most frequent callers to this helpline, making up about 3-quarters of all clients. This finding is in line with helpline use in general [49-51] as well as helplines supporting CSOs in the behavioral health space in particular [31,32]. It also aligns with gender patterns displayed in the literature about CSO support for LOs in other addiction support services [14,52,53] and the amount of time women spend in caregiving activities for their children, compared with men. In the United States, women spend more than twice as much time as men on caregiving activities for a child younger than 18 years [54].

The majority of CSOs contacting the helpline were concerned about a child (1062/1542, 68.9%), although a small but not insignificant percentage were concerned about other LOs such as partners (149/1542, 9.7%), siblings (118/1542, 7.7%), friends (101/1542, 6.5%), parents (73/1542, 4.7%), or grandchildren (39/1542, 2.5%). While much of the information and support shared on the helpline is specifically designed to support a parent-child dyad, there is also a desire for this kind of service for others in the LO’s support system. While parents have a specific kind of leverage to motivate change in an LO [55], siblings, friends, other family members, and chosen family can also benefit from support. Universal information that cuts across all relationships with the LO includes information about substance use and particular substance profiles, communication and opportunities for support, limit-setting, harm reduction, and CSO self-care.

Almost a fifth (3182/17,920, 17.8%) of those contacting the helpline requested services in Spanish, underlining the desire and need for this type of service for Spanish speakers as well as English speakers. Spanish speakers have even fewer outlets for support for an LO and are faced with additional barriers to care such as language, immigration status, and lack of cultural sensitivity. Further research will explore the demographics of Spanish language help-seekers and their unique needs and outcomes.

### Substance

The most frequently cited substance of concern in the sample is cannabis (5266/12,817, 40.9%), followed by opioids (2445/12,817, 19%), including both heroin and prescription painkillers, stimulants (1563/12,817, 12.1%), and alcohol (1286/12,817, 10%). These distributions were moderated by age, with 93.8% of individuals calling about cannabis having LOs younger than 26 years, versus 32.4% of those calling about heroin being younger than 26 years (*P*<.001). These results are not surprising given that the helpline primarily targets parents, and it is possible that family members of older individuals were either not targeted or did not believe they would be served in the same way. Moreover, aside from alcohol, which had a lower incidence in our sample, the results mostly mirror trends in age and primary substance of individuals seeking care and rates of SUD, with cannabis being the highest of any substance [56].

### Substance and Language

Interestingly when comparing English-speaking and Spanish-speaking callers and primary substance of concern, Spanish-speaking CSOs were significantly more likely to call about cannabis and stimulants than English-speaking CSOs. English-speaking CSOs were more likely to be concerned about opioids. It is possible that Spanish-speaking parents are more likely to reach out with concern over cannabis and stimulant use, either because they are more likely to identify their child’s use of this substance, or because they feel that these substances pose an increased risk. Further exploration is needed here to understand the interactions of substance and language among CSOs.

### Use State

In terms of the LO’s use state, the vast majority of CSOs were concerned about an LO who is currently struggling with substances (1231/1753, 70.3%). This is not surprising, given that these LOs are likely demonstrating adverse consequences associated with an SUD which drives help-seeking on the part of the CSO. The need for support here is urgent and immediate, although, for the most part, it does not rise to the level of a 911 call or an Emergency Department visit. On the other hand, the CSO often requires information, emotional support, and help to devise the next steps to bridge the gap between the time of the contact until other support can be put into place—an outpatient visit to a therapist, doctor, or treatment provider, connection to a support group, harm reduction strategies, etc. The helpline often serves as a “stepping stone” to the next part of the journey and helps the CSO create a plan of action. In addition, the strengths-based support and messaging of hopefulness strengthen the CSO’s confidence in being able to navigate this journey.

The prevention group represented only 4.9% (n=88) of the sample, suggesting that not many CSOs will seek human support proactively to prevent a potential substance use problem. These results suggest that reaching these “Prevention” CSOs might be better accomplished via other kinds of services. The recovery group is similarly small (109/1753, 6.2%), perhaps because CSOs who have supported an LO through recovery have other support systems in place, such as after-care services from a treatment center. Alternatively, this could signify a lack of identified need for acquiring tools and skills to support an LO through recovery. Either way, as with the prevention group, this group of “Recovery” CSOs seems to be less likely to seek support via a helpline.

### The Importance of Helplines

The helpline’s free and confidential service reduces frequently cited barriers to care for CSOs such as travel distance/lack of transportation, inconvenient hours of operation, long waits for appointments, financial limitations, and stigma [9,12]. The helpline’s multi-channel service and extended hours reduce the physical effort required to connect, allowing CSOs to reach out from the convenience of their home, car, workplace, or grocery store via a communication channel that feels comfortable for them (eg, phone call, text messaging, or email). Services are available at times that are convenient for working parents—evenings, nights, weekends, and holidays. In addition, help and support via the helpline is available in an immediate, or near-immediate timeframe. Appointments with a medical or mental health provider can take weeks or months to schedule, and CSOs are often in situations that require immediate information and support. Because the helpline service is free, CSOs do not need to struggle with insurance reimbursements or complicated payment structures.

And finally, the service allows for anonymous connection, reducing the concern that a CSO might have about sharing details about this highly stigmatized condition. This last barrier, the stigma that family members carry with them by being associated with someone struggling with substance use [9,11,25,26,57], cannot be overestimated as an inhibiting factor on the part of these CSOs. The helpline service is designed to address this multiplicity of needs—logistical, financial, and emotional. The helpline “meets families where they are,” both literally and figuratively.

Our results show that when this type of service is available, CSOs will reach out. There is a demand for this kind of support from CSOs of all genders, geographic regions, and language preferences. While a range of CSOs connect with the helpline, a few key trends stand out. CSOs are more likely to be parents and women who are concerned about their male children who are 18-25 years old. The greatest demand is around support for cannabis use and for an individual who is struggling and not in substance use treatment.

### Limitations

This analysis had several limitations. First, the variables changed throughout the study. While certain constructs were standardized over time (eg, primary substance of concern and LO age), other variables were only added in later surveys. Some variables remained consistent, while for others, response sets were changed or expanded to better understand participant demographics (eg, LO gender or LO use state). While we attempted to either leave out or combine responses into higher-order variables, this should be noted. Second, some responses were collected by helpline specialists speaking with a CSO over the phone while others were self-reported by the CSO via a web-based survey. While the information is objective (eg, demographics), these differences in data collection may have affected outcomes. Finally, not everyone was required to complete an assessment. As a result, response bias may have affected the data (eg, CSOs concerned about opioid use may have been more or less likely to complete the survey or answer individual survey questions).

Despite these limitations, our data suggest that there is a need for this type of urgent care support among CSOs and that this group will seek out this support when it is available to them. This model should be expanded to helplines such as 988 and other groups where a sizable portion of individuals are seeking support for an LO. Given the mortality and morbidity associated with current substance use, it is necessary to expand the opportunity for more individuals to seek low-burden and low-threshold care.

### Future Directions

There is still a great deal to explore to understand how helpline support can be best used to maximize outcomes for CSOs and their LOs. Areas of future investigation will include several key domains. One area will address the difference between a CSO’s concern over an LO’s substance use versus the actual problem use of that substance. At this point, we caution against making interpretations based on our “primary substance of concern” variable, as this represents the substance that the CSO is most concerned about, not necessarily the substance that is causing the greatest disruption to the LO’s situation. Understanding the difference between “CSO concern” and actual substance-use-related risk will help us build more tailored interventions in the future.

Other areas of further exploration will include understanding how people are finding the helpline, what are the nature of their interactions, and the relationship between demand and communication channels. We will also focus our attention on how CSOs describe their needs and how these needs differ by subgroups such as language, region, race/ethnicity, gender, or sexual orientation. We will seek to understand what CSOs are getting out of the service, using self-reported outcome measurements at 7-day and 90-day intervals post intervention. We will be defining the intervention model, including descriptions of behavioral change techniques and mechanisms of change and how these are linked to both proximal and distal outcomes. Finally, we will discuss how the helpline service fits into the larger ecosystem of care model for CSOs, both at the Partnership to End Addiction and externally.

## Appendix

Multimedia Appendix 1 Additional material.

Multimedia Appendix 2 STROBE (Strengthening the Reporting of Observational Studies in Epidemiology) checklist.

## Abbreviations

CSO: concerned significant other
LGBTQ: lesbian, gay, bisexual, transgender, and queer/questioning
LO: loved one
STROBE: Strengthening the Reporting of Observational Studies in Epidemiology
SUD: substance use disorder

## Data Availability

The datasets analyzed during this study are available from the corresponding author on reasonable request.
