# Supplementary material for: Demographics and Use of an Addiction Helpline for Concerned Significant Others: Observational Study
Source: J Med Internet Res. 2025 Apr 14;27:e55621. doi: 10.2196/55621 (PMC12038293; doi:10.2196/55621)
Supplement: Multimedia Appendix 1 [file jmir_v27i1e55621_app1.docx]

**Multimedia Appendix #1**

The data for this study are from eight datasets collected between April 2011 and December 2021 via four different online platforms: iCarol [1], Typeform [2], SurveyMonkey [3], and Formstack [4]. Between April 2011 and December 2018, 16,088 responses to the iCarol survey were recorded. Between July 2018 and January 2019, 962 responses to four SurveyMonkey surveys were recorded. Between March 2019 and December 2021, 5,292 responses to the Formstack surveys were recorded. Between July 2021 and December 2021, 1,753 responses to the Typeform survey were recorded.

Surveys conducted through iCarol, SurveyMonkey, and Formstack were completed by Helpline specialists based on information provided by the CSO together with the specialist’s input during synchronous telephone calls. The survey on Typeform was filled out via CSO self-report before receiving the Helpline service. Some cases have missing data due to the CSO not providing information, skipping survey questions, or the specialist not entering survey answers. In the case of self-report via Typeform, completion of the assessment is not required to obtain the Helpline service–approximately 20% of those seeking help chose to complete the Typeform survey. These responses are also included in the final aggregated dataset.

The eight surveys are similar, with some minor variations in question phrasing and response sets between versions, primarily on demographic characteristics. Some questions were added in later surveys, e.g. sexual orientation. Questions include information on both the CSO and the LO, including demographic information (gender and geographic location), the language of the interaction, the substance of concern, the CSO’s relationship with the loved one, and the loved one’s use state.

The eight datasets were merged into one aggregated dataset using Stata.^35^ To do so, variables of interest across all the files were matched to variables with similar constructs and combined. Answer choices to these variables were recoded to match each other. When there was variation in response sets between variables, we kept the set with the broader response options. For example, the Formstack response set for a loved one’s frequency of use included once a month or less, 1-3 times/month, 1-3 times/week, 4-6 times/week, and daily. The response set for iCarol is wider, the choices being daily, weekly, and monthly. All options were recoded under the broader set. For qualitative options, the response set with more specific answers would be sorted into and recoded under the more general groups. For example, when there was no corresponding variable across datasets, the individual dataset values were combined and displayed which resulted in large variations in sample size across different variables. The final aggregated sample includes 24,096 total unique responses with the range of responses collected between 2011 to 2021. Descriptive statistics were calculated using IBM SPSS Statistics.^36^.

For variables that were added later, we used the data set with the more comprehensive response set, that would satisfy the question. And secondly, some variables did not have complete response sets in the earlier surveys.

**Table S1.** Data Sources

| Platform | Dates | Number of Responses | Completed By |
| --- | --- | --- | --- |
| iCarol | 4/11-12/18 | 16,088 | Specialist |
| Survey Monkey | 7/18-1/19 | 962 | Specialist |
| Formstack | 3/19-12/21 | 5,292 | Specialist |
| Typeform | 7/21-12/21 | 1,753^a^ | CSO |

^a^Only a subset of CSOs contacting the Helpline have self-report assessment data as it is an optional component of receiving the service.

**References**

1. iCarol. Harris Computer Systems; 2018.
2. Typeform. 2021. Available from: https://typeform.com/
3. SurveyMonkey. Momentiv; 2019. Available from: https://surveymonkey.com/
4. Formstack. 2021. Available from: https://formstack.com/
